# Immunohistochemical analysis of Metadherin in proliferative and cancerous breast tissue

**DOI:** 10.1186/1746-1596-5-38

**Published:** 2010-06-18

**Authors:** Peng Su, Qinghui Zhang, Qifeng Yang

**Affiliations:** 1 Department of Pathology, Qilu Hospital, Shandong University School of Medicine, Ji'nan, China; 2 Department of Breast Surgery, Qilu Hospital, Shandong University School of Medicine, Ji'nan, China

## Abstract

**Background:**

Metadherin (MTDH) has been reported to be associated with cancer progression in various types of human cancers including breast cancer. Whether MTDH contributes to carcinogenesis of breast cancer is still unknown. In the present study, we investigated the expression of MTDH in normal, UDH (usual ductal hyperplasia), ADH (atypical ductal hyperplasia), DCIS (ductal carcinoma in situ) and invasive cancer to explore the possible role of MTDH for breast cancer carcinogenesis.

**Methods:**

Immunohistochemistry was employed on paraffin sections of surgical removed breast samples.

**Results:**

The immunohistochemical results showed almost no staining in normal tissue, moderate staining in ADH and UDH, intense MTDH stains in DCIS and cancer. Statistical analysis demonstrated significant different MTDH expression between proliferative and cancerous breast lesions (*p *< 0.001). MTDH was positively correlated with the histological differentiation of DCIS (*p *= 0.028). In breast cancer, statistical analysis revealed a significant correlation between MTDH expression with patients' age (*p *= 0.042), ER status (*p *= 0.018) and p53 status (*p *= 0.001). We also examined the effect of MTDH on cell proliferation in DCIS and cancer, and we found that MTDH overexpression was significantly correlated with high Ki67 index (*p *= 0.008 and *p *= 0.036, respectively).

**Conclusions:**

MTDH overexpression could be identified in proliferative breast lesions and may contribute to breast cancer progression.

## Background

The intraductal proliferative lesions of breast are a group of cytologically and architecturally diverse proliferations, typically originating from the terminal duct-lobular unit and confined to the mammary duct-lobular system. According to WHO Working Group on Pathology and Genetics of Tumors of the Breast, intraductal proliferative lesions have been divided into usual ductal hyperplasia (UDH), atypical ductal hyperplasia (ADH), flat epithelia atypia (FEA), and ductal carcinoma in situ (DCIS). Clinical studies have indicated UDH, ADH and DCIS in the breast are related to different levels of risk for the subsequent development of invasive carcinoma. The risk factors of subsequent invasive breast carcinoma are 1.5 times for UDH, 4-5 times for ADH, and 8-10 times for DCIS, respectively[1]. Increased interest is to identify factors driving disease progression from UDH, ADH, DCIS to invasive cancer.

Metadherin (MTDH[2], also known as astrocyte elevated gene-1(AEG-1)[3,4], and Lysine-rich CEACAM-1-associated protein(Lyric)[5,6] was originally identified as an oncogene induced in primary human fetal astrocytes infected with human immunodeficiency virus type 1(HIV-1) or treated with HIV envelope glycoprotein(gp120) or tumor necrosis factor-α(TNF-α)[3,7]. Human MTDH/AEG-1 mRNA encodes a 582 amino acid protein with a calculated molecular mass of 64 kDa and pI9.3. It promotes tumourigenesis, metastases and chemoresistance. Several signaling pathways have been found to be associated with the expression of MTDH/AEG-1, including Ha-ras, PI3K/Akt, NF-κB and Wnt/β-catenin[8]. For example, MTDH could cooperate with oncogenic Ha-ras to increase soft agar colony formation of nontumorigenic immortalized melanocytes and HeLa cells[4], also it serves as a downstream target gene of Ha-ras in regulating proliferation and transforming activities[9]. By activating the NF-κB pathway, MTDH could increase anchorage-independent growth and invasiveness of HeLa cells[10]. MTDH also activates cell survival pathways through PI3K/Akt signaling[11]. It has been found that MTDH ubiquitously expresses in numerous cell types, elevated levels have also been observed in some human tumor types, such as breast cancer, prostate cancer, hepatocellular carcinoma, neuroblastoma, esophageal squamous cell carcinoma (ESCC) and non-small cell lung cancer (NSCLC)[12-16]. Expression of MTDH could augment anchorage-independent growth. Overexpression of MTDH could inhibit apoptosis induced by serum starvation in immortalized primary human fetal astrocytes(PHFA). Upregulation of MTDH increased lung metastasis of breast cancer cell, as well as migration and invasion of glioma cells. All these studies suggest that MTDH plays important roles in the oncogenesis of these tumors. Besides the function of oncogenesis, MTDH was also found to be a lipopolysaccharide(LPS)-responsive gene and involved in LPS-induced inflammatory response via NF-κB activation[17].

Although previous studies found that MTDH could mediate lung metastasis of breast cancer[2], serve as a prognostic marker for progression and overall patient survival[13,18], whether MTDH involves in the progression of breast precancerous lesions to cancer is still unknown. In the present study, we focused on elucidating the role of MTDH in the progression of precancerous lesions to breast cancer.

## Methods

### Patients and Tissue samples

This study was conducted on a total of 249 paraffin-embedded breast samples, which were histopathologically diagnosed at department of pathology of Qilu Hospital of Shandong University from 2007 to 2009. The intraductal proliferative lesions included 29 cases of UDH (without atypia), 14 cases of ADH, 37 cases of DCIS including 15 low grade, 7 intermediate grade and 15 high grade. There were 162 cases of invasive ductal carcinoma. Normal breast tissues (n = 7) from reduction mammoplasty specimens were used as a control group. For the use of these clinical materials for research purposes, prior patient content and approval from the Institutional Research Ethics Committee were obtained. All the diagnoses were made following the Pathology and Genetics of Tumors of Breast of World Health Organization Classification of Tumors[1] and were made by two pathologists. Clinicopathologic classification and staging were determined according to the American Joint Committee on Cancer criteria[19].

### Immunohistochemistry

The streptavidin-peroxidase-biotin (SP) immunohistochemical method was performed to study altered protein expression in 249 paraffin-embedded breast tissues. In brief, paraffin-embedded specimens were cut into 4-μm sections and baked at 60°C for 60 min. The sections were deparaffinized with xylenes and rehydrated. Sections were submerged into EDTA antigenic retrieval buffer and microwaved for antigenic retrieval, and then cooled at room temperature for 20 minutes. The sections were treated with 3% hydrogen peroxide in methanol to quench the endogenous peroxidase activity, followed by incubation with normal serum to block nonspecific binding. Rabbit anti-MTDH (1:400; Zymed) was incubated with the sections overnight at 4°C; the second antibody was from SP reagent kit (Zhongshan Biotechnology Company, Beijing, China). After washing, the tissue sections were treated with biotinylated anti-rabbit secondary antibody, followed by further incubation with streptavidin-horseradish peroxidase complex. Stained with diaminobenzidine (DAB), the sections were counterstained with hematoxylin. For negative controls, the rabbit anti-MTDH antibody was replaced with PBS.

### Evaluation of Immunohistochemical Staining

The stained slides were reviewed and scored independently by two observers blinded to the patients' information, and the scores were determined by combining the proportion of positively stained tumor cells and the intensity of staining. Tumor cell proportion was scored as follows:0 (no positive tumor cells); 1 (≤ 20% positive tumor cells); 2 (21-50% positive tumor cells); 3 (51-70% positive tumor cells) and 4 (> 70% positive tumor cells). Staining intensity was graded according to the following criteria: 0 (no staining); 1 (weak staining = light yellow); 2 (moderate staining = yellow brown) and 3 (strong staining = brown). Staining index (SI) was calculated as the product of staining intensity score and the proportion of positive tumor cells. Using this method of assessment, we evaluated MTDH expression in intraductal proliferative lesions to cancer by determining the SI, with scores of 0, 1, 2, 3, 4, 6, 9 or 12. The optimal cutoff value for high and low expression level was identified: an SI score of ≥ 4 was used to define tumors with high MTDH expression, and an SI score of ≤ 3 was used to indicate none or low MTDH expression.

### Statistical Analysis

Analyses were performed using the statistical software package SPSS 13.0 (SPSS, Chicago, IL). The chi-square test or Fisher's exact test were used to evaluate the correlation between MTDH expression and the clinicopathologic characteristics if appropriate. We evaluated the linear-by-linear association from normal, UDH, ADH, DCIS to cancer lesion. Bivariate correlations between study variables were calculated by Spearman's rank correlation coefficients. Differences were considered statistically significant for *p *values < 0.05.

## Results

### Expression of MTDH in UDH, ADH and DCIS

**Figure 1:**
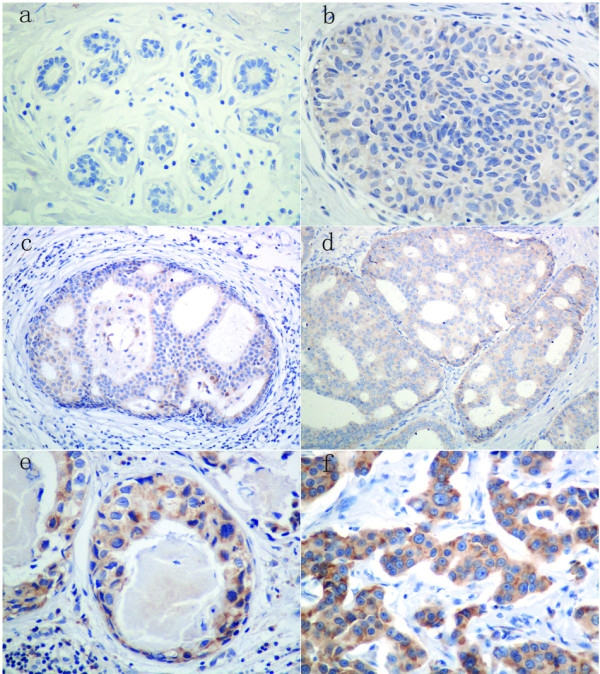
**Expression of MTDH by immunohistochemistry in breast precancerous lesions and cancer**. AEG-1staining was mainly localized in the cytoplasm of cells. a. normal tissue; b. usual ductal hyperplasia (UDH); c. atypical ductal hyperplasia (ADH); d. low grade ductal carcinoma in situ (DCIS); e. high grade ductal carcinoma in situ(DCIS); f. breast cancer.

A positive stain for MTDH was defined as brown stain observed in the cytoplasm (Figure 1). There was no MTDH overexpression identified in normal breast tissue. In the proliferative lesions, overexpression of MTDH protein was expressed in 7/29(24.14%) cases of UDH, 4/14(28.57%) cases of ADH and 27/37(72.97%) cases of DCIS. Furthermore, 90/162(55.56%) cases of invasive breast cancer showed MTDH overexpression. The difference of expression between them was significant tested by linear-by-linear association (*p *< 0.001) (Table 1).

**Table 1:** Expression of MTDH in normal tissues, UDH, ADH, DCIS and breast cancer

Categories	n	MTDH expression			
				
		Low expression	High expression		*p*-value
normal	7	7	0	0.00%	< 0.001
UDH	29	22	7	24.14%	
ADH	14	10	4	28.57%	
DCIS	37	10	27	72.97%	
cancer	162	72	90	55.56%	

*p *value stands for linear-by-linear association

### Relationship of MTDH expression with the status of ER, PR, ErbB-2 and Ki67 in DCIS

There was no significant difference between expression of MTDH and expression levels of universal biological factors such as ER, PR, and ErbB-2 in DCIS. In contrast, statistical analyses indicated that the correlation between MTDH and Ki67 expression was significant (*p *= 0.008) (Table 2), which was further confirmed by Spearman correlation analysis(r = 0.471, *p *= 0.003). We also found the expression in histological differentiation was significant (*p *= 0.028) and the expression difference between the high grade and low grade DCIS was also significant (*p *= 0.035), Spearman correlation of MTDH expression levels to them were 0.379 (*p *= 0.021) and 0.452(*p *= 0.012), respectively. These results suggest that MTDH is overexpressed in highly proliferative DCIS cells and high grade lesions.

**Table 2:** Relationship between MTDH and DCIS

Characteristics	n	MTDH expression		
			
		Low expression	High expression	*p*-value
ER				
negative	7	0	7	0.155
positive	30	10	20	
PR				
negative	9	0	9	0.079
positive	28	10	18	
ErbB-2				
negative	28	10	18	0.079
positive	9	0	9	
Ki67				
low(≤ 10%)	19	9	10	0.008
high(> 10%)	18	1	17	
Histological differentiation				
L&I-DCIS	22	9	13	0.028*
H-DCIS	15	1	14	

* *p*-value between low grade DCIS and high grade DCIS is 0.035

### Relationship of MTDH overexpression with the clinical features of invasive breast cancer

As show in the Table 3, MTDH expression was strongly correlated with the patients' age (*p *= 0.042), Ki67 status (*p *= 0.036), ER status (*p *= 0.018) and p53 status (*p *= 0.001), whereas it was not associated with other clinical characteristics. Spearman correlation analysis was further preformed to confirm the correlation between MTDH expression and patients' age, Ki67, ER and p53 status, which were -0.16(*p *= 0.042), 0.164(*p *= 0.037), -0.185(*p *= 0.018) and 0.261(*p *= 0.001) respectively.

**Table 3:** correlation between MTDH expression and the clinicopathologic characteristics of breast cancer patients

Characteristics	n	MTDH expression		
			
		Low expression	High expression	*p*-value
Age(y)				
≤ 50	66	23	43	0.042
> 50	96	49	47	
Grade				
I&II	118	56	62	0.206
III	44	16	28	
TNM stage				
I	51	25	26	0.607
II	72	32	40	
III	39	15	24	
Tumor size				
T≤ 2 cm	80	39	41	0.276
T > 2 cm	82	33	49	
Lymph node metastasis				
N0	88	40	48	0.778
N1	74	32	42	
Expression of Ki67				
low(≤ 10%)	35	21	14	0.036
high(> 10%)	127	51	76	
Expression of p53				
low	96	53	43	0.001
high	66	19	47	
ER				
negative	66	22	44	0.018
positive	96	50	46	
PR				
negative	64	25	39	0.265
positive	98	47	51	
ErbB-2				
negative	130	61	69	0.201
positive	32	11	21	

## Discussion

With the introduction of mammographic screening, incidence of the precursor lesions has dramatically increased. Pathological and clinical evidence suggests that different intraductal proliferative lesions have different magnitudes of risk for the subsequent development of invasive ductal carcinoma. One of the key challenges is to identify any independent molecular attribute to the transition or as a biomarker to monitor the progression.

Recently, many reports have demonstrated that oncoprotein MTDH is linked to the biological processes such as cancer cell survival, proliferation, apoptosis, migration, metastasis and chemoresistence. MTDH has been also found to be upregulated in several types of human cancers, including breast cancer, prostate cancer, glioblastoma, hepatocellular carcinoma and esophageal carcinoma. All these findings have implicated the role of the overexpression of MTDH in the initiation and progression of cancer. Especially in breast cancer, several reports have found that MTDH could increase lung metastasis of breast cancer cell[2], promote chemoresistance and metastasis by 8q22 genomic gain[18], associate with poor overall survival and promote the proliferation of breast cancer cells through downregulation of tumor suppressor and cell-cycle inhibitor genes, p21^Cip1 ^and p27^Kip1^, possibly via Akt/FOXO1 signaling[13,20]. To investigate whether the expression of MTDH is found in the intraductal proliferative lesions and the upregulation of MTDH is also related to the process of breast oncogenesis, we performed our studies.

In the present study, for the first time, we found that MTDH is low or no expressed in normal cases, but with the intraductal proliferative lesions progression, the expression level and intensity are higher and stronger gradually. This suggests that MTDH might represent a novel indicator for the prognosis of breast lesions and might play a role in the development and progression of breast lesions. We found high expressed MTDH in 72.97% of DCIS, but in 55.56% of invasive ductal carcinoma. This may suggest that MTDH plays a more important role in initiation of ductal carcinoma. We also examined the correlation between MTDH expression and common proliferative marker Ki67 in DCIS and breast cancer. In cancer, the correlation between MTDH and Ki67 is significant (*p *= 0.036), which is in consistent with the result of Li et al[20]. We first found that the correlation is also significant (*p *= 0.008) in DCIS. MTDH is overexpressed in highly proliferative lesions of breast cancer and DCIS. In DCIS, we also found that the expression difference between low grade and high grade (*p *= 0.035). All these might suggest that increased cell proliferation associated with overexpressed MTDH may contribute to the development and progression of breast lesions.

In malignant glioma cells, overexpressed MTDH is located predominantly in the nucleus, where it interacts with the p65 subunit of NF-κB and CBP, thus activating NF-κB signaling[10,21]. MTDH also regulates production of LPS-induced proinflammatory mediators via enhanced NF-κB activation[17]. The transcription factor nuclear factor κB (NF-κB) regulates the expression of a wide variety of genes involved in cellular events such as inflammation, immune response, proliferation and apoptosis[22]. But in our study, we found MTDH mainly localized in the cytoplasm of intraductal proliferative lesions and cancer cells, there is very little nuclear staining. This result is the same with Li et al and Hu et al[13,18], in non-small cell lung cancer, hepatocelluar carcinoma and ESCC the expression also is found mainly in the cytoplasm. So in the proliferative lesions and cancer of breast, MTDH might play an important role, but the exact mechanism is still unknown and need more studies to demonstrate it.

MTDH can induce serum-independent cell growth by blocking serum starvation-induced apoptosis through PI3K-Akt signaling pathways and downstream signaling molecules of Akt including GSK3b-Myc, Bad, MDM2-p53 and p21/mda-6, which cause inhibition of caspase activities[11]. In our present study, we found the correlation between MTDH and p53 expression in protein level using immunohistochemical staining (*p *= 0.001). In invasive cancer, we also found MTDH overexpression was associated with several other poor prognostic features, such as younger patient age (*p *= 0.042) and negative estrogen receptor status (*p *= 0.018). However, our results do not correlate with from findings of Li et al. [13], they reported that there was no correlation found between MTDH expression and patient age or expression levels of estrogen receptors, progesterons receptors. Tumorigenic potential of MTDH was supported by two observations, elevated expression in subsets of cancer cell lines and promotion of anchorage independent growth of immortalized melanocytes and astrocytes[4]. In our study, we found that MTDH expressed in UDH, ADH, DCIS, but the positive cases and intensity were different. (In DCIS, especially in high grade DCIS, the rate of high expression of MTDH is 93.3%; in ADH, the rate is 28.57%; in UDH, the rate is 24.14%).

## Conclusions

Our findings suggest that the overexpression of MTDH may be a useful indicator for intraductal proliferation disease development and progression. Its overexpression is correlated with the proliferation in DCIS and cancer. Further investigation on the molecular mechanism of MTDH involvement in the process of the intraductal proliferative lesions is warranted, especially on the PI3K/Akt and NF-κB signaling pathway.

## Competing interests

The authors declare that they have no competing interests.

## Authors' contributions

PS did the immunohistochemical analysis. QZ reviewed all the pathological slides. QY designed the study and analyzed the data. All authors read and approved the final manuscript
